# Chemical Manipulation; Being Instructions to Students in Chemistry on the Methods of Performing Experiments of Demonstration or Research, with Accuracy and Success

**Published:** 1843-04

**Authors:** 


ART. V.—*Chemical Manipulation; being Instructions to Students in Chemistry on the Methods of performing Experiments of Demonstration or Research, with Accuracy and Success.* By MICHAEL FARADAY, D.C.L. F.R.S. &c. &c. Third Edition.—*London*, 1842. 8vo, pp. 664.

OUR purpose in noticing this important work, is rather to direct the attention of our readers to the fact of its republication, than to express any opinion of its value. For the testimony of the whole scientific world in its favour, putting aside the well-known qualifications of its illustrious author, is a far higher tribute to its merits, than any commendation of ours could be. In first undertaking this work, Mr. Faraday must have been influenced by the simple desire for the promotion of science, by smoothing the practical difficulties in the way of the beginner. He could not expect that the work would add much to his reputation ; nor could he anticipate from it such an amount of pecuniary gain, as should be anything like an adequate compensation for his labour. Highly, therefore, as we have other reasons to think of him as a philosopher, we have found, in the production of this volume, much to increase our admiration of him as a man. To see the pupil and successor of Davy, himself no less illustrious either as a discoverer or expounder of scientific truth, condescending to instruct the tyro in the simplest process of the laboratory, or to explain the newest discoveries in almost any branch of science to an audience of which only a few can be supposed capable of really appreciating them, bespeaks a mind in which the highest talents are united, (would that the combination were less rare,) with the purest and simplest morality.

The present edition of the Chemical Manipulation is little else than a republication of the second, which had been out of print for several years, induced by the continual inquiries made for it. His other avocations, and (as we regret to learn,) his uncertain health, have prevented Mr. Faraday from introducing those changes which the progress of time and of chemical science might seem to make necessary. But, as he justly remarks, however much he might add to the book, he could not take much out : and the various branches of analysis, especially that of organic substances, should rather be treated separately and fully, than slightly hinted at in a preparatory work like the present.

To all who are making the study of chemical science an express object of their pursuit, this volume will afford, we hesitate not to say, that kind of assistance which can only be otherwise obtained by the constant direction and supervention of an accomplished master. It is, in fact, the teaching of Professor Faraday in the Laboratory of the Royal Institution.

